# Identifying the Common Genetic Basis of Antidepressant Response

**DOI:** 10.1016/j.bpsgos.2021.07.008

**Published:** 2021-08-03

**Authors:** Oliver Pain, Karen Hodgson, Vassily Trubetskoy, Stephan Ripke, Victoria S. Marshe, Mark J. Adams, Enda M. Byrne, Adrian I. Campos, Tania Carrillo-Roa, Annamaria Cattaneo, Thomas D. Als, Daniel Souery, Mojca Z. Dernovsek, Chiara Fabbri, Caroline Hayward, Neven Henigsberg, Joanna Hauser, James L. Kennedy, Eric J. Lenze, Glyn Lewis, Daniel J. Müller, Nicholas G. Martin, Benoit H. Mulsant, Ole Mors, Nader Perroud, David J. Porteous, Miguel E. Rentería, Charles F. Reynolds, Marcella Rietschel, Rudolf Uher, Eleanor M. Wigmore, Wolfgang Maier, Naomi R. Wray, Katherine J. Aitchison, Volker Arolt, Bernhard T. Baune, Joanna M. Biernacka, Guido Bondolfi, Katharina Domschke, Masaki Kato, Qingqin S. Li, Yu-Li Liu, Alessandro Serretti, Shih-Jen Tsai, Gustavo Turecki, Richard Weinshilboum, GSRD Consortium, Siegfried Kasper, Joseph Zohar, Daniel Souery, Stuart Montgomery, Diego Albani, Gianluigi Forloni, Panagiotis Ferentinos, Dan Rujescu, Julien Mendlewicz, Major Depressive Disorder Working Group of the Psychiatric Genomics Consortium, Naomi R. Wray, Stephan Ripke, Manuel Mattheisen, Maciej Trzaskowski, Enda M. Byrne, Abdel Abdellaoui, Mark J. Adams, Esben Agerbo, Tracy M. Air, Till F.M. Andlauer, Silviu-Alin Bacanu, Marie Bækvad-Hansen, Aartjan T.F. Beekman, Tim B. Bigdeli, Elisabeth B. Binder, Julien Bryois, Henriette N. Buttenschøn, Jonas Bybjerg-Grauholm, Na Cai, Enrique Castelao, Jane Hvarregaard Christensen, Toni-Kim Clarke, Jonathan R.I. Coleman, Lucía Colodro-Conde, Baptiste Couvy-Duchesne, Nick Craddock, Gregory E. Crawford, Gail Davies, Ian J. Deary, Franziska Degenhardt, Eske M. Derks, Nese Direk, Conor V. Dolan, Erin C. Dunn, Thalia C. Eley, Valentina Escott-Price, Farnush Farhadi Hassan Kiadeh, Hilary K. Finucane, Jerome C. Foo, Andreas J. Forstner, Josef Frank, Héléna A. Gaspar, Michael Gill, Fernando S. Goes, Scott D. Gordon, Jakob Grove, Lynsey S. Hall, Christine Søholm Hansen, Thomas F. Hansen, Stefan Herms, Ian B. Hickie, Per Hoffmann, Georg Homuth, Carsten Horn, Jouke-Jan Hottenga, David M. Hougaard, David M. Howard, Marcus Ising, Rick Jansen, Ian Jones, Lisa A. Jones, Eric Jorgenson, James A. Knowles, Isaac S. Kohane, Julia Kraft, Warren W. Kretzschmar, Zoltán Kutalik, Yihan Li, Penelope A. Lind, Donald J. MacIntyre, Dean F. MacKinnon, Robert M. Maier, Wolfgang Maier, Jonathan Marchini, Hamdi Mbarek, Patrick McGrath, Peter McGuffin, Sarah E. Medland, Divya Mehta, Christel M. Middeldorp, Evelin Mihailov, Yuri Milaneschi, Lili Milani, Francis M. Mondimore, Grant W. Montgomery, Sara Mostafavi, Niamh Mullins, Matthias Nauck, Bernard Ng, Michel G. Nivard, Dale R. Nyholt, Paul F. O’Reilly, Hogni Oskarsson, Michael J. Owen, Jodie N. Painter, Carsten Bøcker Pedersen, Marianne Giørtz Pedersen, Roseann E. Peterson, Wouter J. Peyrot, Giorgio Pistis, Danielle Posthuma, Jorge A. Quiroz, Per Qvist, John P. Rice, Brien P. Riley, Margarita Rivera, Saira Saeed Mirza, Robert Schoevers, Eva C. Schulte, Ling Shen, Jianxin Shi, Stanley I. Shyn, Engilbert Sigurdsson, Grant C.B. Sinnamon, Johannes H. Smit, Daniel J. Smith, Hreinn Stefansson, Stacy Steinberg, Fabian Streit, Jana Strohmaier, Katherine E. Tansey, Henning Teismann, Alexander Teumer, Wesley Thompson, Pippa A. Thomson, Thorgeir E. Thorgeirsson, Matthew Traylor, Jens Treutlein, Vassily Trubetskoy, André G. Uitterlinden, Daniel Umbricht, Sandra Van der Auwera, Albert M. van Hemert, Alexander Viktorin, Peter M. Visscher, Yunpeng Wang, Bradley T. Webb, Shantel Marie Weinsheimer, Jürgen Wellmann, Gonneke Willemsen, Stephanie H. Witt, Yang Wu, Hualin S. Xi, Jian Yang, Futao Zhang, Volker Arolt, Bernhard T. Baune, Klaus Berger, Dorret I. Boomsma, Sven Cichon, Udo Dannlowski, E.J.C. de Geus, J. Raymond DePaulo, Enrico Domenici, Katharina Domschke, Tõnu Esko, Hans J. Grabe, Steven P. Hamilton, Caroline Hayward, Andrew C. Heath, Kenneth S. Kendler, Stefan Kloiber, Glyn Lewis, Qingqin S. Li, Susanne Lucae, Pamela A.F. Madden, Patrik K. Magnusson, Nicholas G. Martin, Andrew M. McIntosh, Andres Metspalu, Ole Mors, Preben Bo Mortensen, Bertram Müller-Myhsok, Merete Nordentoft, Markus M. Nöthen, Michael C. O’Donovan, Sara A. Paciga, Nancy L. Pedersen, Brenda W.J.H. Penninx, Roy H. Perlis, David J. Porteous, James B. Potash, Martin Preisig, Marcella Rietschel, Catherine Schaefer, Thomas G. Schulze, Jordan W. Smoller, Kari Stefansson, Henning Tiemeier, Rudolf Uher, Henry Völzke, Myrna M. Weissman, Thomas Werge, Cathryn M. Lewis, Douglas F. Levinson, Gerome Breen, Anders D. Børglum, Patrick F. Sullivan, Andrew M. McIntosh, Cathryn M. Lewis

**Affiliations:** 1 Department of Psychiatry and Psychotherapy, Medical University Vienna, Austria; 2 Department of Psychiatry, Sheba Medical Center, Tel Hashomer, and Sackler School of Medicine, Tel Aviv University, Israel; 3 Laboratoire de Psychologie Médicale, Université Libre de Bruxelles and Psy Pluriel, Centre Européen de Psychologie Médicale, Brussels; 4 Imperial College School of Medicine, London, UK; 5 Laboratory of Biology of Neurodegenerative Disorders, Neuroscience Department, Istituto di Ricerche Farmacologiche Mario Negri IRCCS, Milan, Italy; 6 Department of Psychiatry, Athens University Medical School, Athens, Greece; 7 University Clinic for Psychiatry, Psychotherapy and Psychosomatic, Martin-Luther-University Halle-Wittenberg, Germany; 8 Université Libre de Bruxelles; 1 Institute for Molecular Bioscience, The University of Queensland, Brisbane, QLD, AU; 2 Queensland Brain Institute, The University of Queensland, Brisbane, QLD, AU; 3 Analytic and Translational Genetics Unit, Massachusetts General Hospital, Boston, MA, US; 4 Department of Psychiatry and Psychotherapy, Universitätsmedizin Berlin Campus Charité Mitte, Berlin, DE; 5 Medical and Population Genetics, Broad Institute, Cambridge, MA, US; 6 Department of Psychiatry, Psychosomatics and Psychotherapy, University of Wurzburg, Wurzburg, DE; 7 Centre for Psychiatry Research, Department of Clinical Neuroscience, Karolinska Institutet, Stockholm, SE; 8 Department of Biomedicine, Aarhus University, Aarhus, DK; 9 Dept of Biological Psychology & EMGO+ Institute for Health and Care Research, Vrije Universiteit Amsterdam, Amsterdam, NL; 10 Division of Psychiatry, University of Edinburgh, Edinburgh, GB; 11 Centre for Integrated Register-based Research, Aarhus University, Aarhus, DK; 12 National Centre for Register-Based Research, Aarhus University, Aarhus, DK; 13 iPSYCH, The Lundbeck Foundation Initiative for Integrative Psychiatric Research, DK; 14 Discipline of Psychiatry, University of Adelaide, Adelaide, SA, AU; 15 Department of Translational Research in Psychiatry, Max Planck Institute of Psychiatry, Munich, DE; 16 Department of Neurology, Klinikum rechts der Isar, Technical University of Munich, Munich, DE; 17 Department of Psychiatry, Virginia Commonwealth University, Richmond, VA, US; 18 Center for Neonatal Screening, Department for Congenital Disorders, Statens Serum Institut, Copenhagen, DK; 19 Department of Psychiatry, Vrije Universiteit Medical Center and GGZ inGeest, Amsterdam, NL; 20 Virginia Institute for Psychiatric and Behavior Genetics, Richmond, VA, US; 21 Department of Psychiatry and Behavioral Sciences, Emory University School of Medicine, Atlanta, GA, US; 22 Department of Medical Epidemiology and Biostatistics, Karolinska Institutet, Stockholm, SE; 23 Department of Clinical Medicine, Translational Neuropsychiatry Unit, Aarhus University, Aarhus, DK; 24 iSEQ, Centre for Integrative Sequencing, Aarhus University, Aarhus, DK; 25 Human Genetics, Wellcome Trust Sanger Institute, Cambridge, GB; 26 Statistical genomics and systems genetics, European Bioinformatics Institute (EMBL-EBI), Cambridge, GB; 27 Department of Psychiatry, Lausanne University Hospital and University of Lausanne, Lausanne, CH; 28 Social, Genetic and Developmental Psychiatry Centre, King’s College London, London, GB; 29 Genetics and Computational Biology, QIMR Berghofer Medical Research Institute, Brisbane, QLD, AU; 30 Centre for Advanced Imaging, The University of Queensland, Brisbane, QLD, AU; 31 Psychological Medicine, Cardiff University, Cardiff, GB; 32 Center for Genomic and Computational Biology, Duke University, Durham, NC, US; 33 Department of Pediatrics, Division of Medical Genetics, Duke University, Durham, NC, US; 34 Centre for Cognitive Ageing and Cognitive Epidemiology, University of Edinburgh, Edinburgh, GB; 35 Institute of Human Genetics, University of Bonn, School of Medicine & University Hospital Bonn, Bonn, DE; 36 Epidemiology, Erasmus MC, Rotterdam, Zuid-Holland, NL; 37 Psychiatry, Dokuz Eylul University School Of Medicine, Izmir, TR; 38 Department of Psychiatry, Massachusetts General Hospital, Boston, MA, US; 39 Psychiatric and Neurodevelopmental Genetics Unit (PNGU), Massachusetts General Hospital, Boston, MA, US; 40 Stanley Center for Psychiatric Research, Broad Institute, Cambridge, MA, US; 41 Neuroscience and Mental Health, Cardiff University, Cardiff, GB; 42 Bioinformatics, University of British Columbia, Vancouver, BC, CA; 43 Department of Epidemiology, Harvard T.H. Chan School of Public Health, Boston, MA, US; 44 Department of Mathematics, Massachusetts Institute of Technology, Cambridge, MA, US; 45 Department of Genetic Epidemiology in Psychiatry, Central Institute of Mental Health, Medical Faculty Mannheim, Heidelberg University, Mannheim, Baden-Württemberg, DE; 46 Department of Psychiatry (UPK), University of Basel, Basel, CH; 47 Department of Biomedicine, University of Basel, Basel, CH; 48 Centre for Human Genetics, University of Marburg, Marburg, DE; 49 Department of Psychiatry, Trinity College Dublin, Dublin, IE; 50 Psychiatry & Behavioral Sciences, Johns Hopkins University, Baltimore, MD, US; 51 Bioinformatics Research Centre, Aarhus University, Aarhus, DK; 52 Institute of Genetic Medicine, Newcastle University, Newcastle upon Tyne, GB; 53 Danish Headache Centre, Department of Neurology, Rigshospitalet, Glostrup, DK; 54 Institute of Biological Psychiatry, Mental Health Center Sct. Hans, Mental Health Services Capital Region of Denmark, Copenhagen, DK; 55 iPSYCH, The Lundbeck Foundation Initiative for Psychiatric Research, Copenhagen, DK; 56 Brain and Mind Centre, University of Sydney, Sydney, NSW, AU; 57 Interfaculty Institute for Genetics and Functional Genomics, Department of Functional Genomics, University Medicine and Ernst Moritz Arndt University Greifswald, Greifswald, Mecklenburg-Vorpommern, DE; 58 Roche Pharmaceutical Research and Early Development, Pharmaceutical Sciences, Roche Innovation Center Basel, F. Hoffmann-La Roche Ltd, Basel, CH; 59 Max Planck Institute of Psychiatry, Munich, DE; 60 MRC Centre for Neuropsychiatric Genetics and Genomics, Cardiff University, Cardiff, GB; 61 Department of Psychological Medicine, University of Worcester, Worcester, GB; 62 Division of Research, Kaiser Permanente Northern California, Oakland, CA, US; 63 Psychiatry & The Behavioral Sciences, University of Southern California, Los Angeles, CA, US; 64 Department of Biomedical Informatics, Harvard Medical School, Boston, MA, US; 65 Department of Medicine, Brigham and Women’s Hospital, Boston, MA, US; 66 Informatics Program, Boston Children’s Hospital, Boston, MA, US; 67 Wellcome Trust Centre for Human Genetics, University of Oxford, Oxford, GB; 68 Institute of Social and Preventive Medicine (IUMSP), Lausanne University Hospital and University of Lausanne, Lausanne, VD, CH; 69 Swiss Institute of Bioinformatics, Lausanne, VD, CH; 70 Division of Psychiatry, Centre for Clinical Brain Sciences, University of Edinburgh, Edinburgh, GB; 71 Mental Health, NHS 24, Glasgow, GB; 72 Department of Psychiatry and Psychotherapy, University of Bonn, Bonn, DE; 73 Statistics, University of Oxford, Oxford, GB; 74 Psychiatry, Columbia University College of Physicians and Surgeons, New York, NY, US; 75 School of Psychology and Counseling, Queensland University of Technology, Brisbane, QLD, AU; 76 Child and Youth Mental Health Service, Children’s Health Queensland Hospital and Health Service, South Brisbane, QLD, AU; 77 Child Health Research Centre, University of Queensland, Brisbane, QLD, AU; 78 Estonian Genome Center, University of Tartu, Tartu, EE; 79 Medical Genetics, University of British Columbia, Vancouver, BC, CA; 80 Statistics, University of British Columbia, Vancouver, BC, CA; 81 DZHK (German Centre for Cardiovascular Research), Partner Site Greifswald, University Medicine, University Medicine Greifswald, Greifswald, Mecklenburg-Vorpommern, DE; 82 Institute of Clinical Chemistry and Laboratory Medicine, University Medicine Greifswald, Greifswald, Mecklenburg-Vorpommern, DE; 83 Institute of Health and Biomedical Innovation, Queensland University of Technology, Brisbane, QLD, AU; 84 Humus, Reykjavik, IS; 85 Virginia Institute for Psychiatric & Behavioral Genetics, Virginia Commonwealth University, Richmond, VA, US; 86 Clinical Genetics, Vrije Universiteit Medical Center, Amsterdam, NL; 87 Complex Trait Genetics, Vrije Universiteit Amsterdam, Amsterdam, NL; 88 Solid Biosciences, Boston, MA, US; 89 Department of Psychiatry, Washington University in Saint Louis School of Medicine, Saint Louis, MO, US; 90 Department of Biochemistry and Molecular Biology II, Institute of Neurosciences, Biomedical Research Center (CIBM), University of Granada, Granada, ES; 91 Department of Psychiatry, University of Groningen, University Medical Center Groningen, Groningen, NL; 92 Department of Psychiatry and Psychotherapy, University Hospital, Ludwig Maximilian University Munich, Munich, DE; 93 Institute of Psychiatric Phenomics and Genomics (IPPG), University Hospital, Ludwig Maximilian University Munich, Munich, DE; 94 Division of Cancer Epidemiology and Genetics, National Cancer Institute, Bethesda, MD, US; 95 Behavioral Health Services, Kaiser Permanente Washington, Seattle, WA, US; 96 Faculty of Medicine, Department of Psychiatry, University of Iceland, Reykjavik, IS; 97 School of Medicine and Dentistry, James Cook University, Townsville, QLD, AU; 98 Institute of Health and Wellbeing, University of Glasgow, Glasgow, GB; 99 deCODE Genetics / Amgen, Reykjavik, IS; 100 College of Biomedical and Life Sciences, Cardiff University, Cardiff, GB; 101 Institute of Epidemiology and Social Medicine, University of Münster, Münster, Nordrhein-Westfalen, DE; 102 Institute for Community Medicine, University Medicine Greifswald, Greifswald, Mecklenburg-Vorpommern, DE; 103 Department of Psychiatry, University of California, San Diego, San Diego, CA, US; 104 KG Jebsen Centre for Psychosis Research, Norway Division of Mental Health and Addiction, Oslo University Hospital, Oslo, NO; 105 Medical Genetics Section, CGEM, IGMM, University of Edinburgh, Edinburgh, GB; 106 Clinical Neurosciences, University of Cambridge, Cambridge, GB; 107 Internal Medicine, Erasmus MC, Rotterdam, Zuid-Holland, NL; 108 Roche Pharmaceutical Research and Early Development, Neuroscience, Ophthalmology and Rare Diseases Discovery & Translational Medicine Area, Roche Innovation Center Basel, F. Hoffmann-La Roche Ltd, Basel, CH; 109 Department of Psychiatry and Psychotherapy, University Medicine Greifswald, Greifswald, Mecklenburg-Vorpommern, DE; 110 Department of Psychiatry, Leiden University Medical Center, Leiden, NL; 111 Virginia Institute for Psychiatric & Behavioral Genetics, Virginia Commonwealth University, Richmond, VA, US; 112 Computational Sciences Center of Emphasis, Pfizer Global Research and Development, Cambridge, MA, US; 113 Institute for Molecular Bioscience Queensland Brain Institute, The University of Queensland, Brisbane, QLD, AU; 114 Department of Psychiatry, University of Münster, Münster, Nordrhein-Westfalen, DE; 115 Department of Psychiatry, Melbourne Medical School, University of Melbourne, Melbourne, AU; 116 Florey Institute for Neuroscience and Mental Health, University of Melbourne, Melbourne, AU; 117 Institute of Medical Genetics and Pathology, University Hospital Basel, University of Basel, Basel, CH; 118 Institute of Neuroscience and Medicine (INM-1), Research Center Juelich, Juelich, DE; 119 Amsterdam Public Health Institute, Vrije Universiteit Medical Center, Amsterdam, NL; 120 Centre for Integrative Biology, Università degli Studi di Trento, Trento, Trentino-Alto Adige, IT; 121 Department of Psychiatry and Psychotherapy, Medical Center - University of Freiburg, Faculty of Medicine, University of Freiburg, Freiburg, DE; 122 Center for NeuroModulation, Faculty of Medicine, University of Freiburg, Freiburg, DE; 123 Psychiatry, Kaiser Permanente Northern California, San Francisco, CA, US; 124 Medical Research Council Human Genetics Unit, Institute of Genetics and Molecular Medicine, University of Edinburgh, Edinburgh, GB; 125 Department of Psychiatry, University of Toronto, Toronto, ON, CA; 126 Centre for Addiction and Mental Health, Toronto, ON, CA; 127 Division of Psychiatry, University College London, London, GB; 128 Neuroscience Therapeutic Area, Janssen Research and Development, LLC, Titusville, NJ, US; 129 Institute of Molecular and Cell Biology, University of Tartu, Tartu, EE; 130 Psychosis Research Unit, Aarhus University Hospital, Risskov, Aarhus, DK; 131 Munich Cluster for Systems Neurology (SyNergy), Munich, DE; 132 University of Liverpool, Liverpool, GB; 133 Mental Health Center Copenhagen, Copenhagen Universtity Hospital, Copenhagen, DK; 134 Human Genetics and Computational Biomedicine, Pfizer Global Research and Development, Groton, CT, US; 135 Psychiatry, Harvard Medical School, Boston, MA, US; 136 Psychiatry, University of Iowa, Iowa City, IA, US; 137 Department of Psychiatry and Behavioral Sciences, Johns Hopkins University, Baltimore, MD, US; 138 Department of Psychiatry and Psychotherapy, University Medical Center Göttingen, Goettingen, Niedersachsen, DE; 139 Human Genetics Branch, NIMH Division of Intramural Research Programs, Bethesda, MD, US; 140 Faculty of Medicine, University of Iceland, Reykjavik, IS; 141 Child and Adolescent Psychiatry, Erasmus MC, Rotterdam, Zuid-Holland, NL; 142 Psychiatry, Erasmus MC, Rotterdam, Zuid-Holland, NL; 143 Psychiatry, Dalhousie University, Halifax, NS, CA; 144 Division of Translational Epidemiology, New York State Psychiatric Institute, New York, NY, US; 145 Department of Clinical Medicine, University of Copenhagen, Copenhagen, DK; 146 Department of Medical & Molecular Genetics, King’s College London, London, GB; 147 Psychiatry & Behavioral Sciences, Stanford University, Stanford, CA, US; 148 NIHR Maudsley Biomedical Research Centre, King’s College London, London, GB; 149 Genetics, University of North Carolina at Chapel Hill, Chapel Hill, NC, US; 150 Psychiatry, University of North Carolina at Chapel Hill, Chapel Hill, NC, US; a Social, Genetic and Developmental Psychiatry Centre, King’s College London, London, United Kingdom; b Department of Medical and Molecular Genetics, King’s College London, London, United Kingdom; c Division of Psychiatry, University College London, London, United Kingdom; d Division of Psychiatry, University of Edinburgh, Edinburgh, United Kingdom; e MRC Human Genetics Unit, University of Edinburgh, Edinburgh, United Kingdom; f Institute of Genetics and Molecular Medicine, University of Edinburgh, Edinburgh, United Kingdom; g Department of Psychiatry and Psychotherapy, Charité – Universitätsmedizin Berlin, corporate member of Freie Universität Berlin, Humboldt-Universität zu Berlin, and Berlin Institute of Health, Department of Medical Psychology, Berlin, Germany; h Department of Translational Research in Psychiatry, Max Planck Institute of Psychiatry, Munich, Germany; i Department of Genetic Epidemiology in Psychiatry, Central Institute of Mental Health, Heidelberg University Faculty of Medicine in Mannheim, Mannheim, Germany; j Department of Psychiatry and Psychotherapy, University of Bonn, Bonn, Germany; k Department of Psychiatry, University of Münster, Münster, Germany; l Department of Psychiatry and Psychotherapy, Medical Center, University of Freiburg, Freiburg, Germany; m Program in Medical and Population Genetics, Broad Institute, Cambridge, Massachusetts; n Analytic and Translational Genetics Unit, Massachusetts General Hospital, Boston, Massachusetts; o Pharmacogenetics Research Clinic, Campbell Family Mental Health Research Institute, Centre for Addiction and Mental Health, Toronto, Ontario, Canada; p Tanenbaum Centre for Pharmacogenetics, Campbell Family Mental Health Research Institute, Centre for Addiction and Mental Health, Toronto, Ontario, Canada; q Division of Adult Neurodevelopment and Geriatric Psychiatry, Centre for Addiction and Mental Health, Toronto, Ontario, Canada; r Institute of Medical Science, Faculty of Medicine, University of Toronto, Toronto, Ontario, Canada; s Department of Psychiatry, Faculty of Medicine, University of Toronto, Toronto, Ontario, Canada; t Department of Psychiatry, Dalhousie University, Halifax, Nova Scotia, Canada; u Department of Psychiatry, Neuroscience and Mental Health Institute, University of Alberta, Edmonton, Alberta, Canada; v Department of Medical Genetics, Neuroscience and Mental Health Institute, University of Alberta, Edmonton, Alberta, Canada; w Department of Psychiatry, Douglas Institute, McGill University, Montreal, Quebec, Canada; x Institute for Molecular Bioscience, University of Queensland, Brisbane, Queensland, Australia; y Department of Genetics and Computational Biology, QIMR Berghofer Medical Research Institute, Brisbane, Queensland, Australia; z Department of Psychiatry, Melbourne Medical School, University of Melbourne, Melbourne, Victoria, Australia; aa Florey Institute for Neuroscience and Mental Health, University of Melbourne, Melbourne, Victoria, Australia; ab Biological Psychiatry Laboratory, IRCCS Fatebenefratelli, Brescia, Italy; ac Department of Biomedical and Neuromotor Sciences, University of Bologna, Bologna, Italy; ad Lundbeck Foundation Initiative for Integrative Psychiatric Research (iPSYCH), Aarhus University, Aarhus, Denmark; ae Department of Biomedicine and Centre for Integrative Sequencing (iSEQ), Aarhus University, Aarhus, Denmark; af Center for Genomics and Personalized Medicine, Central Region Denmark and Aarhus University, Aarhus, Denmark; ag Psychosis Research Unit, Aarhus University Hospital – Psychiatry, Aarhus, Denmark; ah Laboratoire de Psychologie Medicale, Université Libre de Bruxelles, Brussels, Belgium; ai Centre Européen de Psychologie Medicale (PsyPluriel), Brussels, Belgium; aj University Psychiatric Clinic, University of Ljubliana, Ljubljana, Slovakia; ak Department of Psychiatry, Croatian Institute for Brain Research, University of Zagreb Medical School, Zagreb, Croatia; al Psychiatric Genetic Unit, Poznan University of Medical Sciences, Poznan, Poland; am Healthy Mind Lab, Washington University School of Medicine in St. Louis, St. Louis, Missouri; an Department of Psychiatry, Geneva University Hospitals, Geneva, Switzerland; ao Department of Psychiatry, University of Pittsburgh, Pittsburgh, Pennsylvania; ap Department of Psychiatry and Psychology, Mayo Clinic, Rochester, Minnesota; aq Department of Health Sciences Research, Mayo Clinic, Rochester, Minnesota; ar Department of Molecular Pharmacology and Experimental Therapeutics, Mayo Clinic, Rochester, Minnesota; as Department of Neuropsychiatry, Kansai Medical University, Osaka, Japan; at Neuroscience Therapeutic Area, Janssen Research and Development, LLC, Titusville, New Jersey; au Center for Neuropsychiatric Research, National Health Research Institutes, Zhunan, Taipei, Taiwan; av Department of Psychiatry, Taipei Veterans General Hospital, Taipei, Taiwan; aw Division of Psychiatry, School of Medicine, National Yang-Ming University, Taipei, Taiwan; ax Institute of Psychiatry, Psychology and Neuroscience, King’s College London, London, United Kingdom

**Keywords:** Antidepressant response, Depression, Genetics, GWAS, MDD, Polygenic score

## Abstract

**Background:**

Antidepressants are a first-line treatment for depression. However, only a third of individuals experience remission after the first treatment. Common genetic variation, in part, likely regulates antidepressant response, yet the success of previous genome-wide association studies has been limited by sample size. This study performs the largest genetic analysis of prospectively assessed antidepressant response in major depressive disorder to gain insight into the underlying biology and enable out-of-sample prediction.

**Methods:**

Genome-wide analysis of remission (*n*_remit_ = 1852, *n*_nonremit_ = 3299) and percentage improvement (*n* = 5218) was performed. Single nucleotide polymorphism–based heritability was estimated using genome-wide complex trait analysis. Genetic covariance with eight mental health phenotypes was estimated using polygenic scores/AVENGEME. Out-of-sample prediction of antidepressant response polygenic scores was assessed. Gene-level association analysis was performed using MAGMA and transcriptome-wide association study. Tissue, pathway, and drug binding enrichment were estimated using MAGMA.

**Results:**

Neither genome-wide association study identified genome-wide significant associations. Single nucleotide polymorphism–based heritability was significantly different from zero for remission (*h*^2^ = 0.132, SE = 0.056) but not for percentage improvement (*h*^2^ = −0.018, SE = 0.032). Better antidepressant response was negatively associated with genetic risk for schizophrenia and positively associated with genetic propensity for educational attainment. Leave-one-out validation of antidepressant response polygenic scores demonstrated significant evidence of out-of-sample prediction, though results varied in external cohorts. Gene-based analyses identified *ETV4* and *DHX8* as significantly associated with antidepressant response.

**Conclusions:**

This study demonstrates that antidepressant response is influenced by common genetic variation, has a genetic overlap schizophrenia and educational attainment, and provides a useful resource for future research. Larger sample sizes are required to attain the potential of genetics for understanding and predicting antidepressant response.

Major depressive disorder (MDD) is the third leading cause of years lived with disability worldwide (1) and is a substantial risk factor for suicide (2). MDD confers a major personal, societal, and economic burden (3), partly because of the limited efficacy of treatment options.

In 2011 to 2014, 12.7% of individuals in the United States 12 years of age and over reported antidepressant medication use (4). The rate of antidepressant prescriptions is also increasing, with the number of prescriptions doubling in the United Kingdom in the decade prior to 2018 (5). Antidepressants are robustly linked to a reduction in depressive symptoms (6), but they are often ineffective: approximately 35% of patients remit after their primary treatment (7) and approximately 40% develop treatment-resistant depression (TRD), defined as not remitting after two or more antidepressants (8). For patients, the process of trialing antidepressants can be lengthy and demoralizing, delaying recovery and exposing patients to a range of potential side effects that reduce adherence and willingness to try new drugs (9). There is therefore great potential to improve treatment of depression through better understanding of the factors that control response to antidepressants and implementing this knowledge through individually tailored treatment.

Pharmacogenetic studies were expected to uncover loci with large effects on drug response and adverse events due to effects of pharmacokinetic or pharmacodynamic mechanisms. While associations between antidepressant plasma levels and drug-metabolizing enzymes CYP2D6 and CYP2C19 have been identified (10, 11, 12), previous research suggests that genes encoding these enzymes and other candidate genes account for a small proportion of variation in drug response (13,14). However, genotyping complexities for such candidate genes may contribute to limited findings.

Several genome-wide association studies (GWASs) have been performed to identify genetic predictors of antidepressant response. Although no robustly replicated associations have been detected to date (15, 16, 17, 18, 19), common single nucleotide polymorphisms (SNPs) are reported to explain 42% (SE = 18%; 95% confidence interval [CI], 7%–77%) of the variance (20). Pharmacogenetic studies are intensive to perform, requiring disease severity measures at baseline pretreatment and then longitudinally, with many studies being performed as part of a randomized controlled trial (15, 16, 17, 18). This clinically assessed approach provides high-quality data, though it has led to previous studies being limited in sample size, with <3000 patients with MDD in the largest GWAS to date. Further efforts to combine these individual cohorts to increase sample size for genetic studies are therefore required. Use of lighter phenotyping approaches such as electronic health record–derived TRD (21) may also provide novel insight, though it is unclear whether these different measures of antidepressant response have a common genetic basis.

In this study, we analyze genome-wide genetic data on clinically assessed antidepressant response from 5843 patients treated for MDD, combined from 13 international research studies. Using this novel data resource, we perform GWAS of remission and percentage improvement after receiving antidepressant medication, and undertake extensive post-GWAS analyses, made feasible through this increased sample size. This study aims to elucidate the genetic architecture of antidepressant response and use polygenic scores to establish the relationship between antidepressant response and mental health outcomes. We find, for the first time, a replicable polygenic signal of antidepressant response across studies.

## Methods and Materials

### Primary Samples and Measures

This study analyzed 13 cohorts (Table 1). Ten cohorts were of European ancestry and 3 were of East Asian ancestry (Supplement 1). All subjects provided written informed consent for pharmacogenetic analyses. These primary cohorts include individuals with a clinical diagnosis of MDD, who were assessed for depressive symptoms before and after treatment with antidepressants.

**Table 1:** Cohorts of Individuals Diagnosed With Major Depressive Disorder and Assessed for Depressive Symptoms Before and After Treatment With Antidepressant Medication

Study (Reference)	Country, Region	Study Design	Study Length, Weeks	Medication(s)	Measure	Median Age, Years	IQR for Age, Years	Female	*N*^a^	*n*_percentage improvement_	*n*_remit_	*n*_nonremit_
European Ancestry												
STAR∗D (52)	United States	Open label	12	Citalopram	QIDSC	44	32–53	58%	1163	1163	506	657
GSRD (17)	Europe	Naturalistic	>4	Various	MADRS	52.5	43–61	66%	1152	1152	189	963
GENDEP (53)	Europe	Partially randomized RCT	12	Escitalopram, nortriptyline	MADRS	43	33–51	63%	783	783	291	365
DAST (see Supplement 1)	Germany	Naturalistic inpatient	6	Various	HAMD-21	50	37–62	57%	586	586	245	303
PGRN-AMPS (54)	United States	Open label	8	Citalopram, escitalopram	QIDSC	38.5	28–49	63%	490	392	200	290
GENPOD (18)	United Kingdom	Open label	12	Citalopram, reboxetine	BDI	38	30–48	69%	474	474	169	305
PFZ (18)	United States	RCT	6-8	Sertraline, fluoxetine, paroxetine	HAMD-17	43	32–54	67%	309	309	99	210
Mayo (16)	United States	Open label	8	Citalopram, escitalopram	HAMD-17	37	29–51	62%	156	156	80	76
GSK (18)	United States	RCT	8	Escitalopram	HAMD-17	36	25.75–45	55%	132	132	56	76
GODS (18)	Switzerland	Open label	8	Paroxetine	MADRS	37	29.5–43.5	52%	71	71	17	54

East Asian Ancestry												
Miaoli (16)	Taiwan	Open label	8	Escitalopram, paroxetine	HAMD-17	41	30–52	82%	233	233	103	130
Taipei (16)	Taiwan	Open label	8	Fluoxetine, citalopram	HAMD-17	46	34–59	55%	174	174	45	129
Japan (16)	Japan	RCT	6	Fluvoxamine, paroxetine	HAMD-17	44.5	32–56	47%	120	120	78	42
Total									5843	5745	2078	3600

BDI, Beck Depression Inventory; DAST, Depression and Sequence of Treatment; GENDEP, Genome Based Therapeutic Drugs for Depression; GENPOD, GENetic and clinical Predictors Of treatment response in Depression; GODS, Geneva Outpatient Depression Study; GSK, Glaxo Smith Kline; GSRD, Group for the Study of Resistant Depression; HAMD-17, 17-item Hamilton Depression Rating Scale; HAMD-21, 21-item Hamilton Depression Rating Scale; IQR, interquartile range; MADRS, Montgomery–Åsberg Depression Rating Scale; PFZ, Pfizer; PGRN-AMPS, Pharmacogenomics Research Network Antidepressant Medication Pharmacogenomic Study; QIDSC, Quick Inventory of Depressive Symptomatology; RCT, randomized controlled trial; STAR∗D, Sequenced Treatment Alternatives to Relieve Depression.

a Number of participants included after quality control of genetic and clinical data.

Two measures of antidepressant response were defined: remission and percentage improvement. Remission is a binary measure attained when a patient’s depression symptom score decreases to a prespecified threshold for the rating scale (Supplement 1).

All analyses included covariates of the first 20 principal components of population structure, age, and gender. Analyses using the remission measure of response also included the baseline symptom score as a covariate, to control for depression severity.

Each cohort underwent standard quality control and 1000 Genomes Project phase 3 imputation using the RICOPILI pipeline on the LISA server (22) (Supplement 1 and Table S1 in Supplement 2).

### Genome-wide Association Study

GWAS was performed using the RICOPILI pipeline (22) separately for studies with participants of European and of East Asian ancestry (Supplement 1). All other analyses were performed using only the European ancestry cohorts due to the limited sample size of the East Asian cohorts.

### Gene-Level Association Analysis

Gene associations were estimated using MAGMA (23) and transcriptome-wide association study (TWAS) (24).

The MAGMA v1.06b SNP-wise mean model (±10-kb window) was used to perform gene-level association analysis based on the remission and percentage improvement GWAS *p* values. The analysis was based on genetic variants and linkage disequilibrium in the 1000 Genomes Project phase 3 dataset available on the MAGMA website (g1000_eur.bed/bim/fam). SNPs were assigned to genes using the MAGMA NCBI37.3.gene.loc file with a 10-kb window. False discovery rate (FDR) correction was used to control for multiple testing. See Supplement 1 for a description of gene set enrichment analysis using MAGMA.

TWAS integrates GWAS associations with external expression quantitative trait loci data to infer whether differential gene expression estimated from SNP data is associated with the GWAS phenotype. TWAS was performed using FUSION software (http://gusevlab.org/projects/fusion/) and precomputed multi-SNP predictors of gene expression based on data collected from multiple specific brain regions, thyroid tissue, pituitary gland, liver, and blood (Table S2 in Supplement 2). The transcriptome-wide significance threshold of *p* < 2.51 × 10^−6^ was estimated using a permutation procedure (25). To test whether the same causal SNP affects both the GWAS phenotype and gene expression, colocalization analysis was performed using the coloc package in R software (version 3.5.0; R Foundation for Statistical Computing) (26), as implemented by FUSION software.

### Estimation of SNP-Based Heritability

The SNP-based heritability of remission and percentage improvement was estimated using individual-level data by genomic relatedness–based restricted maximum likelihood (GREML) in the software GCTA (genome-wide complex trait analysis) (27,28). The analysis was performed 1) across all cohorts, including a study covariate (mega-GREML); and 2) separately within each cohort and then inverse variance meta-analyzed (meta-GREML) (Supplement 1). Comparison of mega- and meta-GREML estimates can provide insight into the heterogeneity between cohorts, as only mega-GREML accounts for genetic covariances between cohorts. We converted SNP-based heritability estimates for remission to the liability scale using assuming a population prevalence of 0.357, reflecting the prevalence of remission across the cohorts in this study.

### Leave-One-Out Polygenic Scoring

To determine whether polygenic scores derived from the remission and percentage improvement GWAS summary statistics predict antidepressant response in an independent sample, a leave-one-out polygenic scoring approach was used. This involves calculating polygenic scores within each cohort based on GWAS summary statistics derived using all other cohorts. Polygenic scores were calculated using PRSice V2 (29) (Supplement 1). One-sided *p* values were used to assess statistical significance, as we are testing the one-sided hypothesis that the polygenic score has a positive association with the outcome in the target sample.

### Estimation of Genetic Overlap With Mental Health Phenotypes

We tested for evidence of genetic overlap between antidepressant response measures and seven mental health phenotypes: major depression (30), bipolar disorder (31), schizophrenia (32), attention-deficit/hyperactivity disorder (33), autism spectrum disorder (ASD) (34), anxiety (35), and problematic drinking (Alcohol Use Disorders Identification Test problem subscale) (36). Educational attainment (37) was also included, as it has strong correlations with the mental health disorders tested. Evidence of genetic overlap was assessed using polygenic scoring with AVENGEME (38), and linkage disequilibrium score regression (39). To avoid sample overlap between the major depression GWAS and the antidepressant response cohorts in this study, we used major depression GWAS summary statistics excluding overlapping cohorts (STAR∗D [Sequenced Treatment Alternatives to Relieve Depression], GENPOD [GENetic and clinical Predictors Of treatment response in Depression], GENDEP [Genome Based Therapeutic Drugs for Depression], PFZ [Pfizer]).

AVENGEME aggregates polygenic score association results across *p*-value thresholds to estimate genetic covariance between antidepressant response and the eight mental health phenotypes. AVENGEME parameters are provided in Table S3 in Supplement 2. Bonferroni correction was used to account for multiple testing for the eight discovery GWASs used.

### Replication Cohorts and Analyses

#### Out-of-Sample Prediction

External validation of polygenic scores derived using the full GWAS results was also carried out. Five independent samples were used (Supplement 1). In brief, Janssen (*N* = 190, remission rate = 11.8%) (40), the Douglas Biomarker Study (*N* = 127, remission rate = 23.6%) (41), and the IRL-GREY (Incomplete Response in Late Life Depression: Getting to Remission) study (*N* = 307, remission rate = 52.4%) (42) prospectively assessed depressive symptoms, concordant with the discovery GWAS samples. In contrast, Generation Scotland (*n*_treatment resistant_ = 177, *n*_non–treatment resistant_ = 2455) (21) assessed electronic prescription data, and the AGDS (Australian Genetics of Depression Study) study (*n*_responders_ = 4368, *n*_nonresponders_ = 6879) (43) collected retrospective self-report questionnaire data. Polygenic score association results were meta-analyzed across the prospectively assessed cohorts given their more comparable study design and antidepressant measures. One-sided *p* values were used to assess statistical significance.

#### Comparison of Genetic Covariance With Mental Health Phenotypes

Individual-level data were available for Generation Scotland enabling estimation of genetic covariance between TRD and mental health-related phenotypes using AVENGEME, as described above. Analyses in Generation Scotland were controlled for age, gender, and 20 principal components of population structure. When estimating genetic covariance between TRD and major depression, we used major depression GWAS summary statistics excluding Generation Scotland to avoid sample overlap.

## Results

Descriptive statistics for the cohorts used in this study are available in Table 1 and in Figures S1 to S5 in Supplement 1.

### GWAS of Antidepressant Response

Across the 10 European studies, 5151 individuals with remission data (1852 [36.0%] patients remitting) and 5218 participants with percentage improvement data were available. No variants were significantly associated with remission or percentage improvement (Figures S6 and S7 in Supplement 1, Tables S4 and S5 in Supplement 2). There was no evidence of confounding (Figures S8 and S9 in Supplement 1, Table S6 in Supplement 2)

No significant associations were identified in the East Asian GWASs (*N* = 527) (Figures S10 and S11 in Supplement 1). A comparison between East Asian and European GWAS results is shown in Supplement 1.

### Gene-Level Association Results

MAGMA identified a significant association on chromosome 17 for *ETV4* with both remission (*p*_FDR_ = .016) and percentage improvement (*p*_FDR_ = .016). Within the same region, *DHX8* was also significantly associated with remission (*p*_FDR_ = .046). The SNP associations within this region span multiple genes (Figure S12 in Supplement 1). Full MAGMA gene-based association results are shown in Tables S7 and S8 in Supplement 2.

TWAS identified no association achieving transcriptome-wide significance (*p* < 2.51 × 10^−6^). Further inspection of TWAS associations within the chromosome 17 region implicated by MAGMA highlighted SNP-associations with upregulation of *BRCA1* (remission *p* = 1.96 × 10^−4^; percentage improvement *p* = 9.21 × 10^−5^; GTeX brain–caudate [basal ganglia]) and upregulation of *TMEM106A* (remission *p* = .0011; percentage improvement *p* = .0018; Young Finns Study [blood]). Colocalization analysis of these associations indicated shared casual variants for these genes’ differential expression and antidepressant response. Full TWAS results are given in Tables S9 and S10 in Supplement 2.

See Supplement 1 for gene set enrichment analysis results.

### SNP-Based Heritability

**Figure 1:**
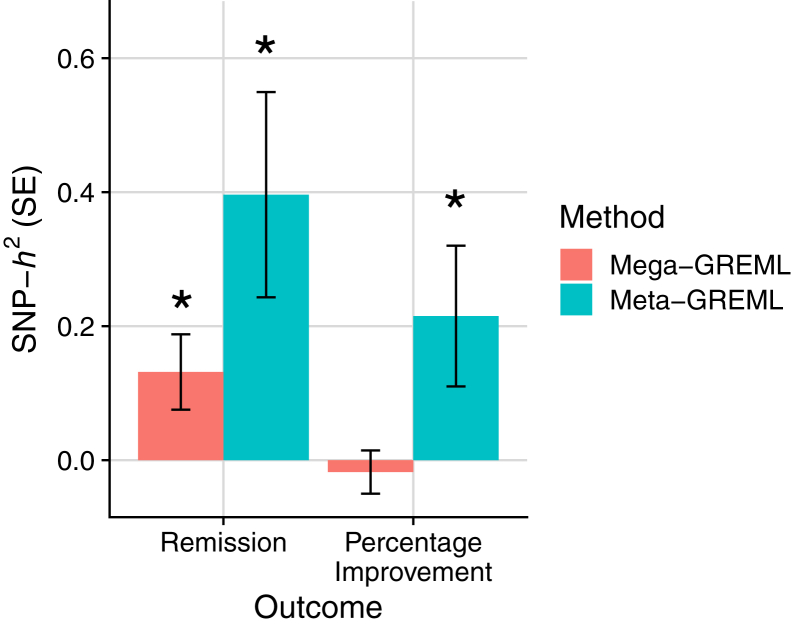
Single nucleotide polymorphism–based heritability (SNP-*h*^2^) estimates for remission and percentage improvement with SE bars. Figure shows across (mega-) and within (meta-) sample genomic relatedness–based restricted maximum likelihood (GREML) estimates. ∗Estimate is significantly different from zero, at *p* < .05.

Analysis across all samples (mega-GREML) showed remission to have a significant nonzero SNP-based heritability (*h*^2^ = 0.132; SE = 0.056; 95% CI, 0.022 to 0.241; *p* = .009, liability scale assuming population prevalence of 0.357), whereas the SNP-based heritability for percentage improvement was not significantly different from zero (*h*^2^ = −0.018; SE = 0.032; 95% CI, −0.080 to 0.045; *p* = .303) (Figure 1).

The SNP-based heritability estimates from meta-analysis of within-sample estimates (meta-GREML) were significant for both remission (*h*^2^ = 0.396; SE = 0.153; 95% CI, 0.096 to 0.696; *p* = .010, liability scale assuming population prevalence of 0.357) and percentage improvement (*h*^2^ = 0.215; SE = 0.105; 95% CI, 0.009 to 0.421; *p* = .041) (Figure 1). See Figures S18 and S19 in Supplement 1 for meta-analysis forest plots.

See Supplement 1 for SNP-based heritability sensitivity analyses.

### Out-of-Sample Prediction

**Figure 2:**
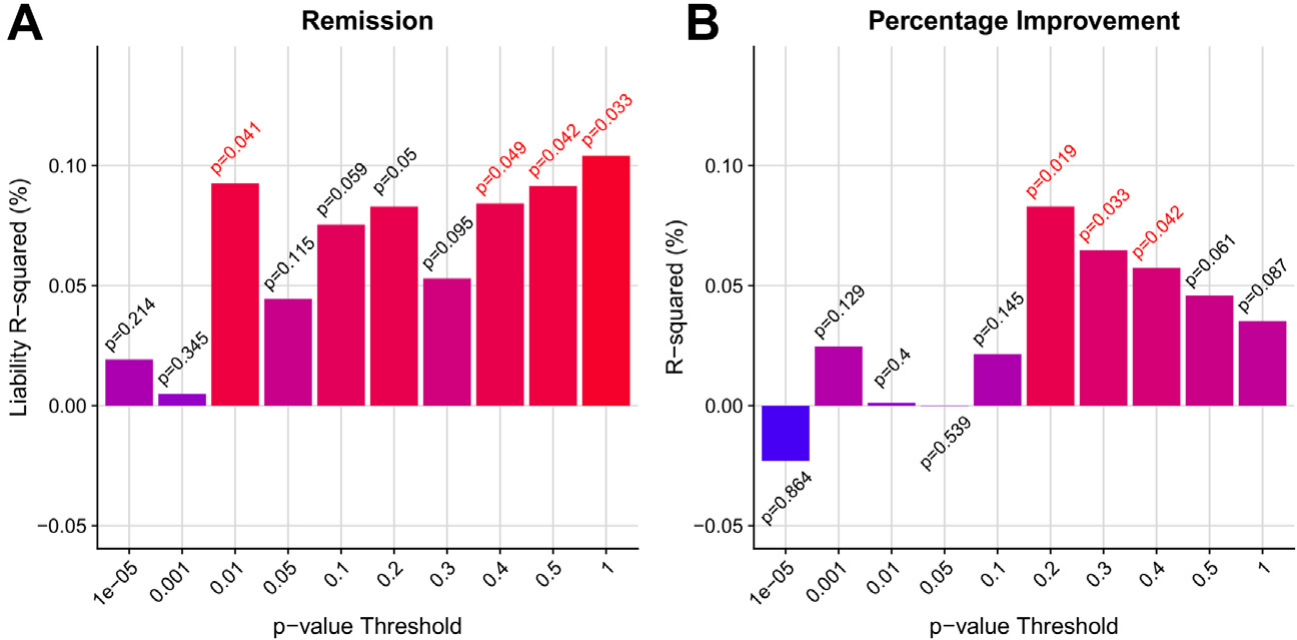
Polygenic prediction of antidepressant response from leave-one-out polygenic scoring for **(A)** remission and **(B)** percentage improvement. *R*^2^ estimates are signed to indicate positive or negative association. One-sided *p* values are shown above or below the bars, with *p* values < .05 highlighted in red.

Leave-one-out polygenic score analysis provided evidence that polygenic scores derived using remission and percentage improvement GWAS results could both explain a statistically significant amount of variance out-of-sample (Figure 2). Both remission and percentage improvement explained ∼0.1% of the variance, with polygenic scores for multiple *p*-value thresholds associated at nominal significance.

Validation of polygenic scores based on the full antidepressant response GWAS summary statistics was carried out using five samples. Meta-analysis of polygenic score associations across the three prospectively assessed cohorts (Janssen, Douglas Biomarker Study, and IRL-GREY study) showed nominally significant evidence of association for the remission polygenic score (maximum liability *R*^2^ = 0.8%, *p* = .015) and a nonsignificant association for the percentage improvement score (maximum *R*^2^ = 0.2%, *p* = .091) (Figure S21 in Supplement 1). Results were highly variable across each prospectively assessed cohort. No association was found between polygenic scores in Generation Scotland or AGDS study cohorts. Full polygenic score replication results are in Tables S14 to S17 in Supplement 2.

### Genetic Overlap With Mental Health Phenotypes

**Figure 3:**
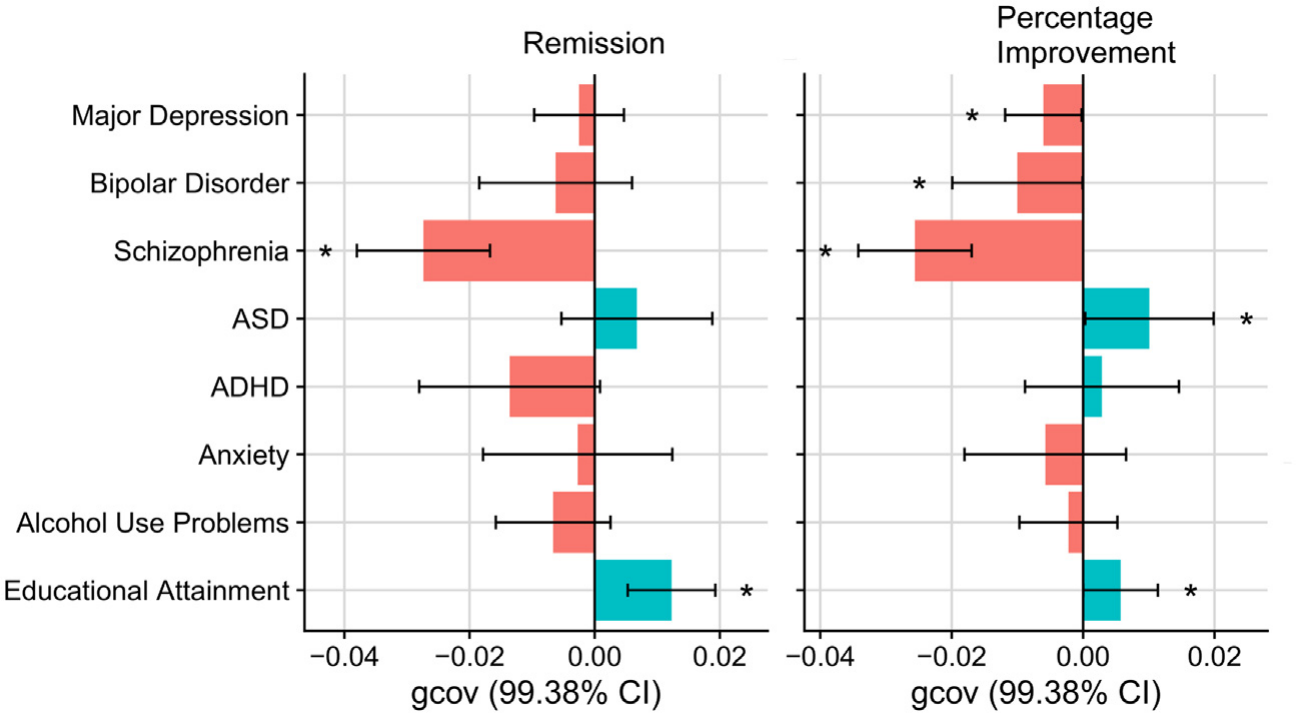
Genetic covariance (gcov) estimates between antidepressant response phenotypes and seven mental health phenotypes and educational attainment. Confidence intervals (CIs) were corrected for multiple testing. ADHD, attention-deficit/hyperactivity disorder; ASD, autism spectrum disorder.

Both remission and percentage improvement showed a significant negative genetic covariance with schizophrenia, and significant positive genetic covariance with educational attainment (Figure 3; Tables S18 and S19 in Supplement 2). Percentage improvement also showed a significant negative covariance with major depression and bipolar disorder, and a significant positive genetic covariance with ASD. Linkage disequilibrium score regression genetic correlation estimates were broadly concordant, although they were nonsignificant (Figure S22 in Supplement 1). Subsequent conditional analysis, covarying for educational attainment polygenic scores, showed that the associations with psychiatric disorders were independent of the association with educational attainment (Figure S23 in Supplement 1).

Genetic overlap estimates between TRD in Generation Scotland and mental health phenotypes were congruent with results from primary samples, showing that genetic risk for schizophrenia was greater among individuals with TRD, and educational attainment genetic propensity was greater among individuals with non-TRD (Figure S24 in Supplement 1).

## Discussion

Antidepressants are a common and effective strategy for treating MDD; however, remission rates are typically low, and factors affecting antidepressant response are poorly understood. This study is the largest genetic investigation of antidepressant response based on clinically defined cohorts. For the first time, we identify a polygenic profile for antidepressant response, which can predict across cohorts, and shows genetic correlations with traits that reflect clinical observations.

This study finds significant evidence that antidepressant response is influenced by common genetic variation. Meta-analysis of SNP-based heritability estimates within each cohort indicates that 20% to 40% of the variance in antidepressant response is attributable to common genetic variation, consistent with a previous analysis of a subset of these studies (20). However, the SNP-based heritability decreased substantially when estimating across cohorts simultaneously. Although the change in SNP-heritability was not statistically significant, these results suggest that antidepressant response in a broad context has a heritable component, but genetic differences can explain additional variability in antidepressant response within more specific contexts. Despite the apparent heterogeneity across individual cohorts, the sample sizes for antidepressant response are sufficiently large to detect a polygenic signal. Genetic studies for susceptibility to psychiatric disorders show that findings accrue after an inflection point in sample size is reached (30, 31, 32). This study’s findings for SNP-based heritability and out-of-sample polygenic prediction indicate that sample sizes for antidepressant response are reaching the inflection point and that larger studies will uncover more of the genetic component (44). Power calculations for detecting genome-wide significant variation, and the variance explained by corresponding polygenic scores, are provided in Figure S25 in Supplement 1. Interestingly, our findings suggest that the SNP-based heritability of remission is higher than for percentage improvement. The percentage improvement score might have lower heritability because of increased noise, in which this measure is more susceptible to random variation in depressive symptoms, is less comparable across the different depressive symptom scales used, or captures increases in depressive symptoms.

This study provides novel insight into the shared genetic basis between antidepressant response and mental health phenotypes. We show an association between high genetic liability of psychiatric disorders and poorer response, which mirrors conclusions of clinical studies (45). The schizophrenia polygenic risk score was negatively associated with antidepressant response, which is replicated in the TRD phenotype in Generation Scotland. Previous studies have shown that individuals with TRD may respond to antipsychotic medication (46). Our findings extend those reports by suggesting that individuals with antidepressant resistance also have a higher burden of schizophrenia genetic risk. We found some evidence that genetic liability to major depression is associated with poorer response to antidepressants. However, this association was only statistically significant for percentage improvement, and it requires replication. In addition, we report a novel finding that high ASD genetic liability increased the chance of remission. Another recent study reported that ASD genetic liability is associated with poorer response to cognitive behavioral therapy (47). If both these findings are replicated, it would suggest ASD genetic liability could serve as a differential predictor of response to antidepressants and cognitive behavioral therapy. We also identified a significant association between genetic propensity for educational attainment and improved antidepressant response as well as between genetic propensity for educational attainment and non-TRD. This may reflect the indirect measurement of socioeconomic status captured by educational attainment, which is supported by previous literature showing a positive association between antidepressant response and socioeconomic status (48). Future research should explore whether individuals with higher educational attainment have improved response due to factors such as adherence or joint psychological treatment.

Polygenic scores derived from the remission and percentage improvement GWASs both significantly predicted antidepressant response out of sample using a leave-one-out design. This is the first GWAS of antidepressant response able to predict significantly out of sample, representing an important advance in the field of antidepressant response genetics. Although the variance explained is low (*R*^2^ = 0.1%) and *p* values are close to the nominal significance threshold, this result is encouraging given the sample size of this study. For example, a recent GWAS of MDD explains only 1.9% of the variance in MDD, despite having a sample size 100 times greater than this study (30). Our finding suggests that a renewed effort to systematically collect new samples in which genetic associations with antidepressant response can be identified will improve the prediction of antidepressant response, helping to uncover its biological mechanisms and clinical associations, and eventually enable more accurate clinical predictors to be developed and applied.

This study provided limited insight into the biological underpinnings of antidepressant response implicating one locus on chromosome 17 surrounding *ETV4* and *DHX8*. A previous study using neuronal cell lines and mouse models found that *ETV4* mediates brain-derived neurotrophic factor (*BDNF*) induced hippocampal dendrite development and plasticity (49), congruent with the hypothesis that the mechanism of action for antidepressants is via hippocampal neuroplasticity (50). *DHX8* has a less clear mechanistic link to antidepressant response with a broader function in messenger RNA splicing (51). Replication of the association at this locus is required before further experimental investigation.

In addition, no association was detected with genetic variation within classical pharmacokinetic candidate genes, such as *CYP2D6* and *CYP2C19*, which have previously been robustly associated with antidepressant plasma levels (11). Although the enzymatic activity of CYP2D6 and CYP2C19 is largely regulated by common genetic variation, these variants include structural variants that are not well captured by GWAS arrays, and large effects on enzymatic activity are typically conferred by combinations of genetic variants (haplotypes), which GWAS does not assess. Therefore, the absence of an association at this point may be a false negative result. Furthermore, looking across individuals that have not been treated with a specific antidepressant or antidepressant class will reduce the likelihood of detecting pharmacokinetic effects.

Owing to a limited sample size, it was not possible to estimate genetic correlations between longitudinally assessed antidepressant response and TRD defined using electronic health records. However, comparison of shared genetic etiology with other mental health phenotypes indicated that these distinct measures of antidepressant response have a shared genetic basis. Further comparison and integration of these two approaches is warranted and may prove fruitful given the large gains in sample size that electronic health record–derived phenotypes can provide.

There are several limitations to this study that should be addressed in the future. First, large sample sizes are essential for robust identification of associated genetic variation and out-of-sample prediction. However, combining independently collected datasets inevitably introduces heterogeneity. Obtaining large homogeneous samples is particularly challenging for pharmacogenetic studies, as heterogeneity is driven not only by patient characteristics such as diagnosis and patient ascertainment, but also by differences in treatment such as the drug, dosage, duration, and co-pharmacotherapy. Although the cohorts within this study have many features in common, heterogeneity in antidepressant treatment is present. As sample sizes grow, analyses stratified by these factors will become more feasible, enabling detection of genetic effects relevant to each antidepressant, antidepressant class, or other treatment characteristics. Second, an important question to consider is whether the variance in depressive symptoms after treatment is due to antidepressant response or to other variables altering the course of depression. Although antidepressants have a significant effect on depressive symptoms, and their administration is the core feature of participants in this study, individuals may vary in depressive symptoms due to other factors affecting disease progression, such as clinical and sociodemographic variables and placebo response. This is a difficult issue to resolve but should be considered when interpreting the results. Future genetic studies incorporating the placebo arm of clinical trials may help identify genetic associations specific to antidepressant response. Third, this study has focused on changes in total depressive symptoms without considering symptom domain-specific changes or the presence of side effects. Given the wide range of depressive symptoms and the influence side effects can have on efficacy, consideration of these features may provide additional insights. Fourth, although this study included three cohorts of East Asian ancestry, further inclusion of cohorts with diverse ancestries is an important area. Genetic analysis within diverse populations helps to ensure that the findings are applicable to worldwide populations and can help fine-map causal variants underlying genetic associations.

In summary, this study identifies a polygenic profile for antidepressant response that predicts across studies and is negatively correlated with genetic susceptibility to schizophrenia, which could be used for prognostic purposes. While the current results have no clinical utility as a pharmacogenetic test, they indicate that studies with larger sample sizes could provide predictions explaining a substantial proportion of antidepressant response. We note that a prognostic test that enables even a modest increase in the proportion of patients that respond to antidepressants would have a substantial impact on recovery for many patients, given the high prevalence of depression. We hope that this study prompts both replication and extension to accelerate the development of pharmacogenetic testing for psychiatry.

## Supplementary Material

Supplement 2

Key Resources Table
